# Dermoscopy of Melanoma and Non-melanoma Skin Cancers

**DOI:** 10.3389/fmed.2019.00180

**Published:** 2019-08-21

**Authors:** Junji Kato, Kohei Horimoto, Sayuri Sato, Tomoyuki Minowa, Hisashi Uhara

**Affiliations:** Department of Dermatology, Sapporo Medical University School of Medicine, Sapporo, Japan

**Keywords:** dermoscopy, melanoma, basal cell carcinoma, sebaceous carcinoma, actinic keratosis, Bowen's disease, squamous cell carcinoma, Merkel cell carcinoma

## Abstract

Dermoscopy is a widely used non-invasive technique for diagnosing skin tumors. In melanocytic tumors, e.g., melanoma and basal cell carcinoma (BCC), the effectiveness of dermoscopic examination has been fully established over the past two decades. Moreover, dermoscopy has been used to diagnose non-melanocytic tumors. Here, we review novel findings from recent reports concerning dermoscopy of melanoma and non-melanoma skin cancers including BCC, sebaceous carcinoma, actinic keratosis, Bowen's disease, squamous cell carcinoma (SCC), Merkel cell carcinoma (MCC), extramammary Paget's disease (EMPD), and angiosarcoma.

## Introduction

Dermoscopy is a non-invasive technique for diagnosing skin lesions, which aids in the differentiation between benign and malignant alterations. Dermoscopy has been used to diagnose non-melanocytic tumors. Recently, vascular morphology has been identified as an important criteria in dermoscopic diagnosis when assessing non-melanocytic tumors. Here, we review recent reports of novel findings related to dermoscopy of melanoma and non-melanoma skin cancers.

### Melanoma

Dermoscopy has been shown to improve significantly the diagnosis of melanocytic lesions in the clinical practice. Following the Consensus Net Meeting on Dermoscopy in 2000, a two-step algorithm for a method of dermoscopic diagnostic was established, especially for pigmented skin lesions (1). Marghoob and Braun subsequently devised a revised two-step algorithm including polarized dermoscopy and blood vessel morphology (2). In the second step of the revised algorithm, pattern analysis, the ABCD rule, the Menzies method (3), and the seven-point checklist are employed to diagnose melanoma. The seven-point checklist is based on seven melanoma-specific criteria; each is classified as major or minor. Major criteria consist of atypical networks, blue-whitish veils, and atypical vascular patterns while minor criteria are irregular dots/globules, irregular streaks, irregular blotches, and regression structures. A score of 2 is given to each of the major criteria, whereas a score of 1 is given to each of the minor criteria. Results yielding 3 points or more should be considered suspicious enough to justify exclusion.

### Lentigo Maligna and Lentigo Maligna Melanoma

The differential diagnoses related to lentigo maligna (LM) and lentigo maligna melanoma (LMM) include a myriad of other pigmented skin lesions, including solar lentigo, seborrheic keratosis, and pigmented actinic keratosis. While the pigment network is the dermoscopic hallmark of superficial spreading melanoma (SSM) located on the trunk and extremities, a true pigment network is rarely found in LM. A pseudonetwork pattern refers to the common dermoscopic finding of melanocytic and non-melanocytic pigmented macules of the face; it is a structureless diffuse brown pigmentation interrupted by numerous, variably broad, and hypopigmented holes, which correspond to hair follicles and sweat gland openings. Based on a report by Schiffner et al., the four most important features are asymmetric pigmented follicular openings, dark rhomboidal structures, slate-gray globules, and slate-gray dots. This analysis yields a sensitivity of 89% and a specificity of 96% (4).

### Nodular Melanoma

Considering that most dermoscopic features have been described in the context of SMM, the dermoscopic recognition of nodular melanoma (NM) is also challenging since the tumor often lacks the well-known melanoma-specific criteria. Therefore, several accepted dermoscopic criteria of melanoma are not detected in purely nodular tumors.

Argenziano et al. described a new predictor of NM, namely the presence of blue-black color within the lesion (5). The blue-black color is thought to reflect the combination of pigments localized in the mid-deep dermis (blue) and the epidermis (black). The authors reported that all lesion surfaces that were comprised of at least 10% blue and black areas were significantly associated with pigmented NM. Moreover, Pizzichetta et al. reported NM was related to features such as ulceration, homogeneous disorganized patterns, homogeneous blue-pigmented structureless areas, multiple (≥3) colors, the combination of polymorphous vessels and milky-red globules/areas, and symmetric shapes (6).

### Acral Melanoma (Volar)

Saida et al. reported dermoscopic patterns of acral melanoma in the Japanese population (7). They found that the parallel ridge pattern (PRP) is the most specific dermoscopic finding for acral melanomas. PRP consists of a band-like pigmentation located on the ridges of the skin markings. In their report, the sensitivity and specificity of PRP for acral melanoma were 86.4 and 99%, respectively. In additional studies, Phan et al. and Ozdemir et al. reported that PRP was detectable in 53 and 60.8% of their cases (8, 9). Lallas et al. proposed the BRAAFF checklist, a score composed of four positive features, i.e., irregular blotches, PRP, asymmetry of structures, and asymmetry of colors, and two negative features such as the parallel furrow pattern (PFP) and fibrillar pattern. Based on the results of the BRAAFF checklist, they proposed a dermoscopic diagnostic algorithm that achieves 93.1% sensitivity and 86.7% specificity for acral melanoma diagnosis (10).

It is difficult to differentiate melanoma from melanocytic nevus if PRP or PFP is not observed. In melanoma on the volar skin without typical dermoscopic patterns, Mikoshiba et al. recently reported frequently observed features consisting of asymmetry, greater numbers of colors (≥3), irregular distribution of blue-white structures, dots and globules, vascular structures including milky red areas, ulcers, diffuse pigmentation, and irregular streaks (11).

### Nail Apparatus (Subungual) Melanoma

Important dermoscopic features of nail apparatus (so-called subungual) melanoma include irregular lines on a brown background, micro-Hutchinson's sign triangular pigmentation on the nail plate, and a wide pigmented band (12). The width of pigmentation is an important risk factor. Ohn et al. reported the following factors to be associated with adult-onset *in situ* lesions: width of pigmentation ≥3 mm, width of pigmentation ≥6 mm (the minimum 6-mm width was more strongly associated with melanoma), multicolor pigmentation, asymmetry, border fading, and the Hutchinson sign (13). Moreover, Benati et al. reported that a width more than 2/3 of the nail plate suggested melanoma (14). They also reported that the second most important predictor of nail apparatus melanoma was the presence of gray to black color. This gray to black color was associated with 12.5% of nevus and 76% of nail apparatus melanoma.

Clinically, age is important to differentiate melanoma from melanocytic nevus. Most cases of nail apparatus melanoma are diagnosed in adults. Caution must be observed before diagnosing nail apparatus melanoma in children because irregular findings are frequently observed in the benign nevus of nails in children.

### Amelanotic Melanoma

Amelanotic melanomas are a relatively rare subtype of melanomas with little or no pigment at visual inspection. Since the majority of melanoma-associated dermoscopic structures are pigmented, amelanotic melanomas remain a demanding diagnostic. The atypical vascular structures are frequently the only clue for its diagnosis (15, 16). Therefore, diagnosis and treatment are often delayed. Menzies et al. reported that the dermoscopically diagnostic accuracy of hypomelanotic or amelanotic melanomas was inferior to that of pigmented melanoma (15). In a recent study of amelanotic melanomas, Lin et al. reported polymorphous vessels as common features in 20 of 27 melanomas. However, in truly amelanotic melanomas, the vascular pattern of polymorphous vessels may not suffice as an only feature utilized to diagnose amelanotic melanoma since it is also found in other skin cancers (16).

### Basal Cell Carcinoma

The value of dermoscopy of basal cell carcinoma (BCC) has been extensively demonstrated over the past few decades. Menzies et al. reported the sensitivity of diagnostic criteria for pigmented BCC was 97% (17). The dermoscopic criteria associated with BCCs include the absence of a pigment network and the presence of specific features, e.g., arborizing vessels, large blue-gray ovoid nests, multiple blue-gray globules, leaf-like areas, spoke wheel areas, and ulceration. In reports from Menzies et al. and Altamura et al., arborizing vessels (52% in Menzies et al., 48.5% in Altamura et al.), large blue-gray ovoid nests (55%, 60.8%), multiple blue-gray globules (27%, 38.5%), leaf-like areas (17%, 22.7%), spoke wheel areas (10%, 13.1%), and ulceration (27%, 34.6%) were dermoscopically observed in pigmented BCCs (17, 18). Additional features have been reported recently, specifically multiple small erosions, shiny white streaks, and concentric structures (19–23). Altamura et al. reported that multiple small erosions were seen in 14.1% of non-pigmented BCCs (18), whereas shiny white streaks have been seen only in polarized dermoscopy (22, 24). Moreover, vascular patterns such as short fine telangiectasias (SFTs), arborizing microvessels, and milky-pink backgrounds have been reported, and these patterns may be useful particularly for non-pigmented BCCs. SFTs are small vessels without branches. Micantonio et al. (20) and Emiroglu et al. (25) reported that SFTs were the second most common vascular pattern found in BCCs. These telangiectasias were significantly more common in superficial BCCs than in nodular BCCs (20). Additionally, Pan et al. reported that arborizing microvessels, short, bright red, sharply focused, fine-caliber branching vessels, were seen in 62% of superficial BCCs (26). Reports on milky-pink backgrounds indicated that they were more common in superficial BCCs (21, 25–27).

Dermoscopic characteristics for each BCC subtype have been described. Some reports differentiated between superficial BCC and other subtypes by dermoscopy (19, 24, 25). In superficial BCC, maple leaf-like areas, spoke wheel areas, SFTs, multiple small erosions, and concentric structures were frequently observed (17–19, 23). Ahnlide et al. (23) summarized dermoscopic features in the common subtype of BCC, including superficial BCC (*n* = 202), nodular BCC (*n* = 76), and infiltrated BCC (*n* = 142) (Table 1). Based on their reports, both in nodular and infiltrated BCCs, arborizing vessels were the most common, while ulcerations were the second most common findings. Moreover, Lallas et al. reported that blue-gray ovoid nests may be predictors for non-superficial BCCs. According to their findings, both arborizing vessels and ulcerations would exclude superficial BCC (19).

**Table 1:** Dermoscopic features in subtype of BCC in the report of Ahnlide et al. (23).

	**Nodular BCC (%)**	**Superficial BCC (%)**	**Infiltrated BCC (%)**
Arborizing vessels	79.7	28.6	86.5
Blue-gray ovoid nests	28.9	21.4	18.3
Ulceration	35.3	20	58.7
Leaf-like areas/spoke wheel areas	16	37.1	50.8
Multiple small erosions	10.7	47.1	15.1
Shiny red-white, structureless areas	25.1	55.7	29.4

### Sebaceous Carcinoma

Although the common clinical presentation of sebaceous carcinoma (SC) is a yellow or red nodule, clinical diagnosis is challenging due to the absence of criteria for dermoscopic diagnosis (28, 29). The main dermoscopic features of SC are yellowish structures, polymorphous vessels, ulcerations, and whitish-pink areas (30–36) (Table 2). The most common findings are yellowish structures, i.e., background, globules and yellow/yellowish areas, which were described as the heterogeneously distributing yellowish objects with varying size, shape and number in the literatures. Polymorphous vessels and ulceration are the second most common appearances. Polymorphous vessels were reported as the combination of various types of vessels, i.e., linear irregular vessels, hairpin vessels, comma-like vessels, dotted vessels, arborizing vessels, and telangiectasias. Ulcerations or crusts on the surface of the tumor suggest the possibility of malignancy, which might help distinguish SC from the benign sebaceous tumors. Whitish-pink areas were described as the homogeneous white-to-pink background (35). Dermoscopically, benign sebaceous tumors should be differentiated from SC. Benign sebaceous tumors are classified as sebaceous hyperplasia, sebaceous adenoma, and sebaceoma (31). Sebaceous hyperplasia consists of a white-yellow, umbilicated, structureless center, radially arranged yellow globules, and peripheral telangiectasias (crown vessels). The absence of polymorphous vessels and ulcerations might be helpful to differentiate from SC.

**Table 2:** Dermoscopic features in sebaceous carcinoma.

**References**	**Number of cases**	**Dermoscopic findings**
Coates et al. (30)	2	Variably yellow background Polymorphous vessels Ulceration
Lallas et al. (31)	2	Yellowish structure White-yellowish well-circumscribed areas Polymorphous vascular pattern Ulceration
Manríquez et al. (32)	2	Yellow globules Multiple telangiectasia
Iikawa et al. (33)	1	Homogeneous yellow background Polymorphous vessels
Satomura et al. (34)	5	Yellow background Polymorphous vessels
Horimoto et al. (35)	3	Pink-whitish areas Yellow-whitish globules Polymorphous vessels Ulceration
Nair et al. (36)	1	Yellowish background with peripheral vessels Ulceration

### Actinic Keratosis

A grading classification of actinic keratosis (AK) has been proposed based on clinical criteria (37). Grade I (mild) consists of slightly palpable AK, grade II (moderate) exhibits readily palpable and visible AK and grade III (severe) is characterized by very thick and hyperkeratotic AK. This clinical classification of AK corresponds to dermoscopic characteristics (38–42). In grade I, red pseudonetwork patterns and white scales are seen. In grade II, a strawberry pattern is typical. The strawberry pattern results from an erythematous background with white-to-yellow keratotic and dilated follicular openings. In addition, targetoid hair follicles are also seen in grade II (43). In grade III, either enlarged follicular plugs filled with keratotic plugs over a white to yellow background or marked hyperkeratosis are observed.

AKs cannot always be distinguished from SCC *in situ* or invasive SCC. Vascular patterns may be helpful concerning this. Casari et al. reported that the increasing atypia is usually associated with dotted vessels around follicles in severe AKs (44). Therefore, enlarging dotted vessels, forming glomerular vessels were seen *in situ* SCC. With the progression to invasive SCC, hairpin and/or linear-irregular vessels will appear.

In pigmented AK, the most typical dermoscopic structure is the superficial brown network consisting of brown, curved double lines that surround enlarged, partially confluent, keratotic follicles of various sizes. Pigmented AK may also reveal an annular-granular pattern and pseudonetwork and rhomboidal structures, which are also seen in lentigo maligna (45). The annular-granular pattern in pigmented AK tends to be more prominent and keratin may be observed in the follicular openings. Moreover, the strawberry sign, rosette pattern, and scale may help to distinguish AK from LM when making a diagnosis (43).

### Bowen's Disease

The dermoscopic characteristics of Bowen's disease (BD) were first described in 2004 (46).

Based on several studies, glomerular vessels (69–97%) and scaly white-to-yellow surfaces (64–96%) were commonly observed in non-pigmented BD (26, 46–50). Brown to gray globules/dots and structureless pigmentation were observed in 21–80% and 70–78% of pigmented BD, respectively (46, 48, 50, 51). These pigmented globules/dots are often distributed at the periphery, sometimes exhibiting a streak-like or leaf-like structure. However, these are not specific to pigmented BD. Chung et al. reported that streak-like structures did not converge toward the center of the lesion, nor did they connect to a common base and therefore may be distinguished from those seen in melanocytic lesions or in BCCs (52). Recently, Yang et al. found two new dermoscopic signs by analyzing 146 lesions of BD (49). One was parallel pigmented edges at the periphery of the lesion, named the double-edge sign. The other was several aggregated large pigmented massive structures, often distributed at the periphery of the lesion, named clusters of brown structureless areas. The former was observed in 44 of 146 lesions (30.1%), and the latter was in 56 (38.4%).

In rare occasions, BD develops in the nail, periungually, in the fingers and palm, often displaying the type of pigmented BD (53–55). Nakayama et al. reported four cases of periungual pigmented BD (53). Histologically, dilated vessels in the papillary dermis corresponding to a dermoscopic finding of glomerular vessels were observed, but none of the four cases presented glomerular vessels upon dermoscopic examination. They speculated that acral skin has a thicker stratum corneum than other skin, so papillary vessels might not be detectable by dermoscopy. In addition, the PFP was found in two of four cases, while the PRP was not found in any case. Similarly, Cavicchini et al. reported a case of pigmented BD developing in the palm that showed a PFP (54). The presence of the scaly surface and the lack of the PRP may distinguish acral BD from acral melanoma. Regarding BD developed in the fingernails, several case reports described subungual BD presented longitudinal melanonychia (55–57). Additionally, Hutchinson's sign was also observed in some reports (53, 58, 59).

### Squamous Cell Carcinoma

Dermoscopic criteria for squamous cell carcinoma (SCC) include the presence of keratin/scales, blood spots, white circles, white structureless areas, hairpin vessels, linear-irregular vessels perivascular white halos, and ulceration (39, 60, 61). Keratin and scales are homogeneous opaque yellow to brown structures, corresponding to hyperkeratosis and parakeratosis (60, 62). Blood spots are the multiple red to black dots in the keratin mass, corresponding to small crusts or hemangiomas (60). White circles are the bright white circles surrounding a dilated infundibulum corresponding to acanthosis and hypergranulosis of the infundibular epidermis (62). White structureless areas are the whitish areas covering large areas of tumors, corresponding to large targetoid hair follicles (39, 63). Among the criteria discussed above, keratin and white circles reached the sensitivity and specificity for SCC diagnostic at a rate of 79 and 87%, respectively (60). The presence of vessels in more than half of the tumor's surface with a diffuse distribution of vessels and bleeding significantly increased the possibility of poorly differentiated SCC. Conversely, keratin/scales are a potent predictor of well- and moderately differentiated SCC (64). However, Pyne et al. reported that moderately and poorly differentiated SCC displayed more branched and serpentine vessels than well-differentiated SCC and that moderately and poorly differentiated SCC displayed larger numbers of vessel types than well-differentiated SCC (61). Regarding lip SCC, Benati et al. recently reported that scales, white structureless areas, and white halos were observed in the majority of the cases (100, 91, and 86%, respectively) (65).

### Merkel Cell Carcinoma

Several recent studies have defined useful significant dermoscopic features for Merkel cell carcinoma (MCC). The most common dermoscopic finding is the milky-red areas that are usually associated with linear irregular vessels. Jalilian et al. reported dermoscopic features of 12 MCC cases (66). All cases presented polymorphous, linear irregular vessels, and structureless areas. Milky-pink areas were observed in 11 cases. Furthermore, Harting et al. reported that milky-red areas or linear irregular vessels were most commonly observed in 10 MCC cases studies (67). Additionally, Dalle et al. observed milky-red areas in 80% of their patients. While these patterns may be observed in amelanotic melanoma, the lack of findings of pigmentation or blue-gray veils could direct diagnosis against MCC (68).

### Extramammary Paget's Disease

Dermoscopic features of extramammary Paget's disease (EMPD) have not yet been established due to its rarity. Recently, Mun et al. compared the dermoscopic appearance of 35 EMPD cases and EMPD-mimicking lesions, e.g., eczematous dermatitis (ED), fungal infections (FI), and Bowen's disease (BD). In EMDP, they observed milky-red areas (32/35), dotted vessels (18/35), glomerular vessels (7/35), polymorphous vessels (6/35), surface scales (7/35), linear irregular vessels (1/35), ulcers/erosions (15/35), pigmented structures (11/35), shiny white lines (4/35), and white structureless areas (3/35) (69). Milky red areas were significantly more prevalent in EMPD than in ED, FI, or BD. Moreover, invasive EMPD correlated statistically with polymorphous vessels.

### Angiosarcoma

Dermoscopic features of angiosarcoma (AS) have not yet been established due to its rarity. De Giorgi et al. described steam-like areas with a white or skin-colored central area as a characteristic dermoscopic feature of AS (70). Furthermore, Oiso et al. reported that a gradation of various colors within the lesion may be an important dermoscopic feature of AS since it is not present in common purpura or ecchymosis (71). Minagawa et al. reported that AS is characterized by the absence of well-defined vascular structures, e.g., lacunae/lagoons that are commonly found in other vascular lesions such as angioma and pyogenic granuloma. Those features might be useful toward differential diagnosis with amelanotic melanomas (72).

## Conclusion

We summarized recent reports of novel findings related to dermoscopy of melanoma and non-melanoma skin cancers. Dermoscopy is presently thought to be effective and helpful for diagnosing melanoma and non-melanoma skin cancers. However, it is important to consider that dermoscopy is just one of several means, others being clinical history, age and gross appearance, that can be utilized in cancer diagnosis. Therefore, we should not hesitate to do a biopsy in cases in which a diagnosis cannot be reached clearly through dermoscopy.

## Author Contributions

JK and HU have full responsibility of this article. JK, KH, SS, TM, and HU confirmed the manuscript for submission.

### Conflict of Interest Statement

The authors declare that the research was conducted in the absence of any commercial or financial relationships that could be construed as a potential conflict of interest.
